# Loss of *miR‐34* in *Drosophila* dysregulates protein translation and protein turnover in the aging brain

**DOI:** 10.1111/acel.13559

**Published:** 2022-02-15

**Authors:** Ananth R. Srinivasan, Tracy T. Tran, Nancy M. Bonini

**Affiliations:** ^1^ Department of Biology University of Pennsylvania Philadelphia Pennsylvania USA

**Keywords:** aging, autophagy, *miR‐34*, neurodegeneration, proteostasis, translation

## Abstract

Aging is a risk factor for neurodegenerative disease, but precise mechanisms that influence this relationship are still under investigation. Work in *Drosophila melanogaster* identified the microRNA *miR*‐*34* as a modifier of aging and neurodegeneration in the brain. *MiR*‐*34* mutants present aspects of early aging, including reduced lifespan, neurodegeneration, and a buildup of the repressive histone mark H3K27me3. To better understand how *miR*‐*34* regulated pathways contribute to age‐associated phenotypes in the brain, here we transcriptionally profiled the *miR*‐*34* mutant brain. This identified that genes associated with translation are dysregulated in the *miR*‐*34* mutant. The brains of these animals show increased translation activity, accumulation of protein aggregation markers, and altered autophagy activity. To determine if altered H3K27me3 was responsible for this proteostasis dysregulation, we studied the effects of increased H3K27me3 by mutating the histone demethylase *Utx*. Reduced *Utx* activity enhanced neurodegeneration and mimicked the protein accumulation seen in *miR*‐*34* mutant brains. However, unlike the *miR*‐*34* mutant, *Utx* mutant brains did not show similar altered autophagy or translation activity, suggesting that additional *miR*‐*34*‐targeted pathways are involved. Transcriptional analysis of predicted *miR*‐*34* targets identified *Lst8*, a subunit of Tor Complex 1 (TORC1), as a potential target. We confirmed that *miR*‐*34* regulates the 3’ UTR of *Lst8* and identified several additional predicted *miR*‐*34* targets that may be critical for maintaining proteostasis and brain health. Together, these results present novel understanding of the brain and the role of the conserved miRNA *miR*‐*34* in impacting proteostasis in the brain with age.

## Graphical abstract

The highly conserved microRNA *miR‐34* regulates regulates two distinct pathways that influence proteostasis in the fly brain. *miR‐34* targets components of the PRC2 complex and a subunit of TORC1. In the absence of *miR‐34*, these pathways become misregulated, leading to altered proteostasis.

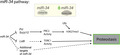

## 1 INTRODUCTION

Brain aging is associated with susceptibility to neurological disease and is one of the most prominent risk factors for neurodegeneration. Studying the cellular processes that change with age provides valuable insight into how aging can increase the risk of neurodegeneration. For example, changes in protein regulation with age, such as protein misfolding or aggregation, can cause cellular toxicity and tissue degeneration. Likewise, changes in transcriptional regulation with age, such as chromatin remodeling or aberrant gene expression, can drive disease susceptibility. In studying the basic biology of aging, researchers have defined key effectors and pathways that modulate aging, brain health, and neurotoxicity.

MicroRNAs are small non‐coding RNAs that post‐transcriptionally regulate gene expression and represent a well‐established class of modifiers that influence brain health (Filipowicz et al., 2008). MicroRNAs have also been implicated in regulating aging and neurodegeneration (Bilen et al., 2006; Karres et al., 2007; Lee et al., 2008). Of note, the conserved microRNA *miR*‐*34* was discovered to be uniquely upregulated in the fly brain with age and is a modulator of lifespan and brain health (Liu et al., 2012). Loss of *miR*‐*34* leads to age‐associated neurodegenerative effects, such as brain tissue degeneration and a brain transcriptional profile reflective of that of an older animal (Liu et al., 2012). Conversely, upregulation of *miR*‐*34* is potently protective against degeneration and suppresses the formation of protein accumulations of the pathogenic polyglutamine protein (SCA3trQ78) of Spinocerebellar Ataxia type 3 (SCA3). Additional studies into the *miR*‐*34* pathway identified two novel targets, *Pcl* and *Su(z)12*, that regulate neurotoxicity. Mutations in either target mimic, in part, the protective effect of *miR*‐*34* upregulation against SCA3 toxicity (Kennerdell et al., 2018), defining a paradigm by which *miR*‐*34* promotes brain health.

Both Pcl and Su(z)12 are subunits of the Polycomb Repressive Complex 2 (PRC2) that places the tri‐methyl mark on Lysine 27 of Histone 3 (H3K27me3). H3K27me3 is a repressive histone mark that normally increases with age (Booth & Brunet, 2016). In *miR*‐*34* mutants, these PRC2 subunits are upregulated and animals have increased levels of H3K27me3 in the brain with age and a reduced lifespan (Kennerdell et al., 2018). Conversely, mutations in PRC2 subunits globally decrease H3K27me3 and extend lifespan of flies (Ma et al., 2018), suggesting that modulation of this histone mark regulates pathways implicated in aging.

As individual microRNAs regulate many targets, the range of cellular functions regulated by *miR*‐*34* is still unclear and additional pathways regulated by *miR*‐*34* may also play an important role in aging and brain degeneration. Therefore, we better characterized pathways altered in the aged *miR*‐*34* mutant brain compared to the normal aging brain. We identify a new role for *miR*‐*34* in regulating proteostasis in the fly brain. The previously identified *miR*‐*34*‐regulated H3K27me3 pathway modulates some, but not all, proteostasis impairments observed in the mutant. We suggest that *miR*‐*34* targets additional transcripts involved in brain proteostasis, and identify *Lst8*, a subunit of Tor Complex 1 (TORC1), as a target of *miR*‐*34*. These findings further our understanding of the role of *miR*‐*34* in maintaining brain health and provide new context for future studies on *miR*‐*34*, its targets, and its role in brain aging.

## 2 RESULTS

### 2.1 Transcriptomic analysis of *miR*‐*34* mutant brain highlights dysregulated proteostasis and translation

**FIGURE 1:**
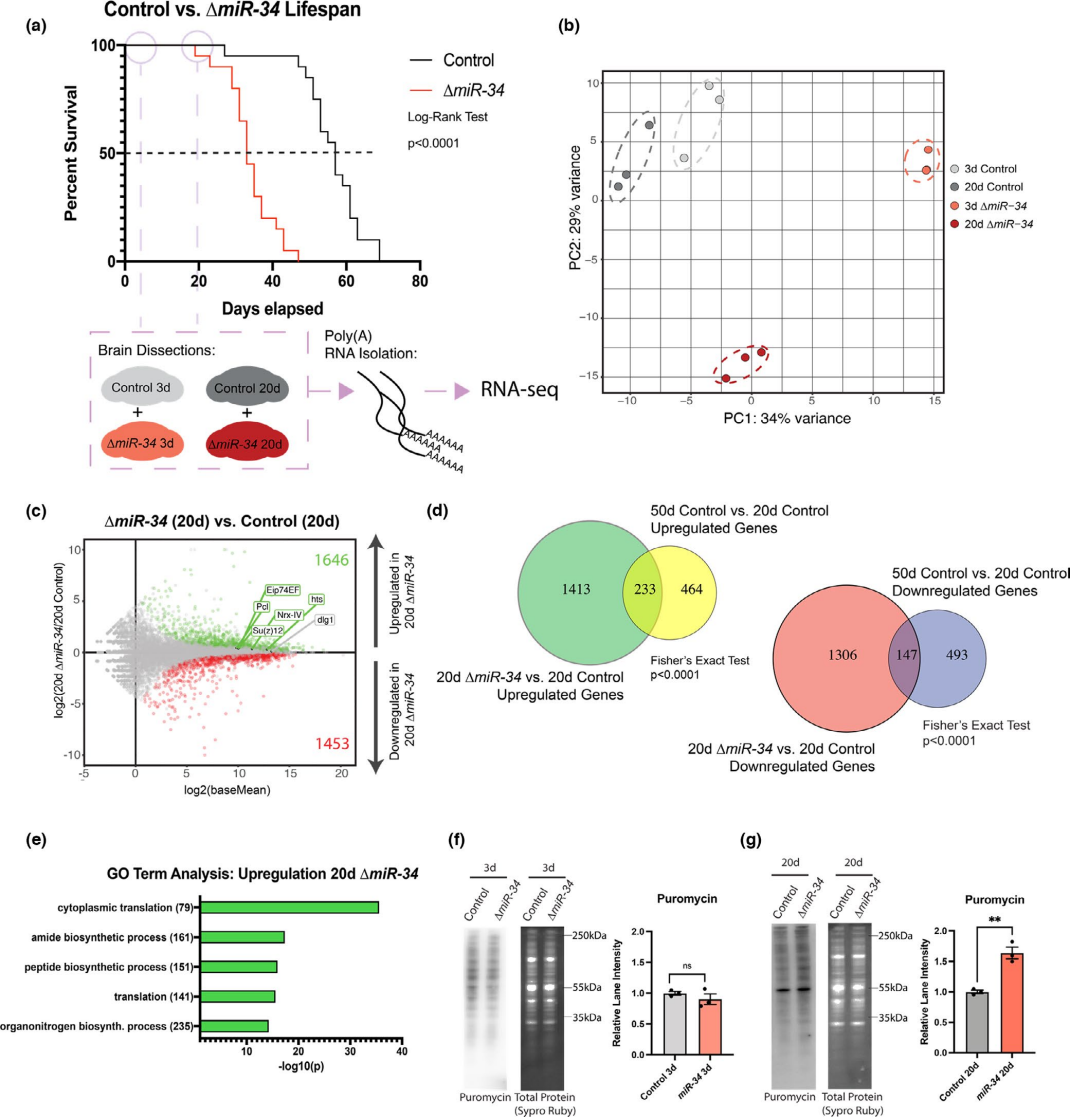
*miR*‐*34* mutants have increased translation activity in the brain with age. (a) Lifespan curve highlighting 3d and 20d RNA‐seq time points for control and *miR*‐*34* mutant animals (*n* = 120 flies/condition). (b) Principal component analysis of transcriptome time points for control and *miR*‐*34* mutant brains (3d, 20d). (c) MA plot showing differential gene expression for 20d *miR*‐*34* vs. control brains. Green dots represent genes upregulated in *miR*‐*34* mutant (*p*_adj _< 0.05). Red dots represent genes downregulated in the *miR*‐*34* mutant (*p*_adj _< 0.05). Six validated targets of *miR*‐*34* are highlighted. (d) Venn diagram showing significant overlap between genes that are significantly upregulated (left, 5.25‐fold over‐enriched) and significantly downregulated (right, 3.37‐fold over‐enriched) in 20d *miR*‐*34* vs. control brain and genes up or downregulation in 50d vs. 20d control brains. (e) Gene ontology (GO) term analysis for significantly upregulated genes (20d *miR*‐*34* vs. control) showing top 5 pathways. (f) Western immunoblots of puromycin‐labeled protein in control and *miR*‐*34* mutant brains (3d). No significant difference (n=3 biological replicates, mean ± SEM, Student's *t* test). (g) Western immunoblots of puromycin‐labeled proteins in control and *miR*‐*34* mutant brains (20d). *miR*‐*34* mutants have a higher level of puromycin‐labeled protein. (n=3 biological replicates, mean ± SEM, Student's *t* test). Significance: **p < 0.01

To better define *miR*‐*34* pathways that may impact health and brain aging, we examined gene expression changes in the brains of *miR*‐*34* mutant and control animals. Brains of young (3d) and older (20d) control and *miR*‐*34* mutant animals were dissected for polyA+RNA sequencing (Figure 1a). Principal component analysis (PCA) showed that *miR*‐*34* mutant brains presented unique transcriptional profiles at both 3d and 20d relative to each other and control brains (Figure 1b). These findings indicate that the loss of *miR*‐*34* leads to distinct transcriptional changes in the fly brain of both young and older animals.

We identified differentially expressed genes between *miR*‐*34* mutant brains and age‐matched controls. Significant differentially expressed genes were defined using a Benjamini–Hochberg *p*_adj_<0.05 cutoff (Figure 1b, Figure S1a, File S1). Six TargetscanFly database predicted targets (Agarwal et al., 2015; Agarwal, 2018) of *Drosophila miR*‐*34* have been validated to date (Kennerdell et al., 2018; Liu et al., 2012; McNeill et al., 2020; Xiong et al., 2016). Five of these targets were significantly upregulated at 20d in the *miR*‐*34* mutant brain, and all six were also significantly upregulated in the 3d brain (Figure 1c, Figure S1a), consistent with the loss of *miR*‐*34* in these brains.

These data indicated that the 20d aged *miR*‐*34* mutant brain had striking changes in gene expression relative to its age‐matched control at a time near the inflection point for *miR*‐*34* on its lifespan curve. We were curious if the changes at this time point would be similar to changes seen near the mortality inflection point of control animals, such as in a 50d old brain. Thus, an additional RNA‐sequencing experiment was performed on control brains of 3d, 20d, and 50d. We first identified gene changes between 20d and 50d control brains to determine whether there were similarities between gene changes with age and the gene changes of 20d‐*34* mutant vs. 20d control brains (Figure S1b, c, File S2). These analyses showed a significant overlap in upregulated and downregulated genes between the two groups (Fisher's exact test, *p *< 0.0001) (Figure 1d). Using the 3d time point, we also defined genes that consistently changed with age (Figure S1b). These genes had a significant overlap with gene changes in the 20d *miR*‐*34* mutant (vs. age‐matched control) (Figure S1d) and were enriched for similar GO Terms as genes in the 50d control and 20d *miR*‐*34* (vs. 20d control brains) groups (File S3). Together, these data indicate that 20d *miR*‐*34* mutants share some gene expression similarities with natural brain aging, but also have a distinct transcriptional profile.

Roughly ~30% of all significantly altered genes in the 50d (vs. 20d) control brain were also altered in the same manner in the 20d mutant (vs. 20d control) brain. Surprisingly, however, there were ~2.3‐fold more gene changes in the 20d mutant than 50d control brain (Figure 1d). These data suggest that the loss of *miR*‐*34* is driving many additional cellular changes that expand beyond those that occur with natural aging. We were motivated to understand if these changes reduced brain health with age, to define pathways that explain how *miR*‐*34* function may be crucial for brain aging and health. GO Term analysis on all significantly up‐ and downregulated genes between the 20d mutant vs. 20d control brain highlighted biological processes most altered in the *miR*‐*34* mutant (Figure 1e, Figure S1e). “Cytoplasmic Translation” was the most significant GO Term among upregulated genes (Figure 1e). This GO term was specific to the aged *miR*‐*34* mutant brain and was not enriched among genes upregulated in 3d mutant brain or 50d aged brain conditions (Figure S1g, File S3). Thus, the loss of *miR*‐*34* appears to uniquely lead to aberrant translation activity with age.

To assess a functional consequence of translation‐related genes, we examined protein translation activity in the *miR*‐*34* mutant brain. A puromycin feeding assay (Schmidt et al., 2009; Zamurrad et al., 2018) was used to determine if *miR*‐*34* mutant brains showed altered protein synthesis. Young (3d) control vs. 3d *miR*‐*34* mutant brains showed similar levels of puromycin‐labeled protein (Figure 1f). Aged (20d) *miR*‐*34* mutant brains, however, had increased puromycin‐labeled protein with no change in feeding behavior (Figure 1g). The gain in puromycin‐labeled protein along with the transcriptional changes in the 20d mutant brain suggests that the upregulation of translation genes in the aged *miR*‐*34* mutant brain correlates with a significant increase in translation activity. These results also underscored that the loss of *miR*‐*34* leads to changes in protein homeostasis.

### 2.2 ***MiR*‐*34*** **mutants exhibit increased protein accumulation in the brain with age**

**FIGURE 2:**
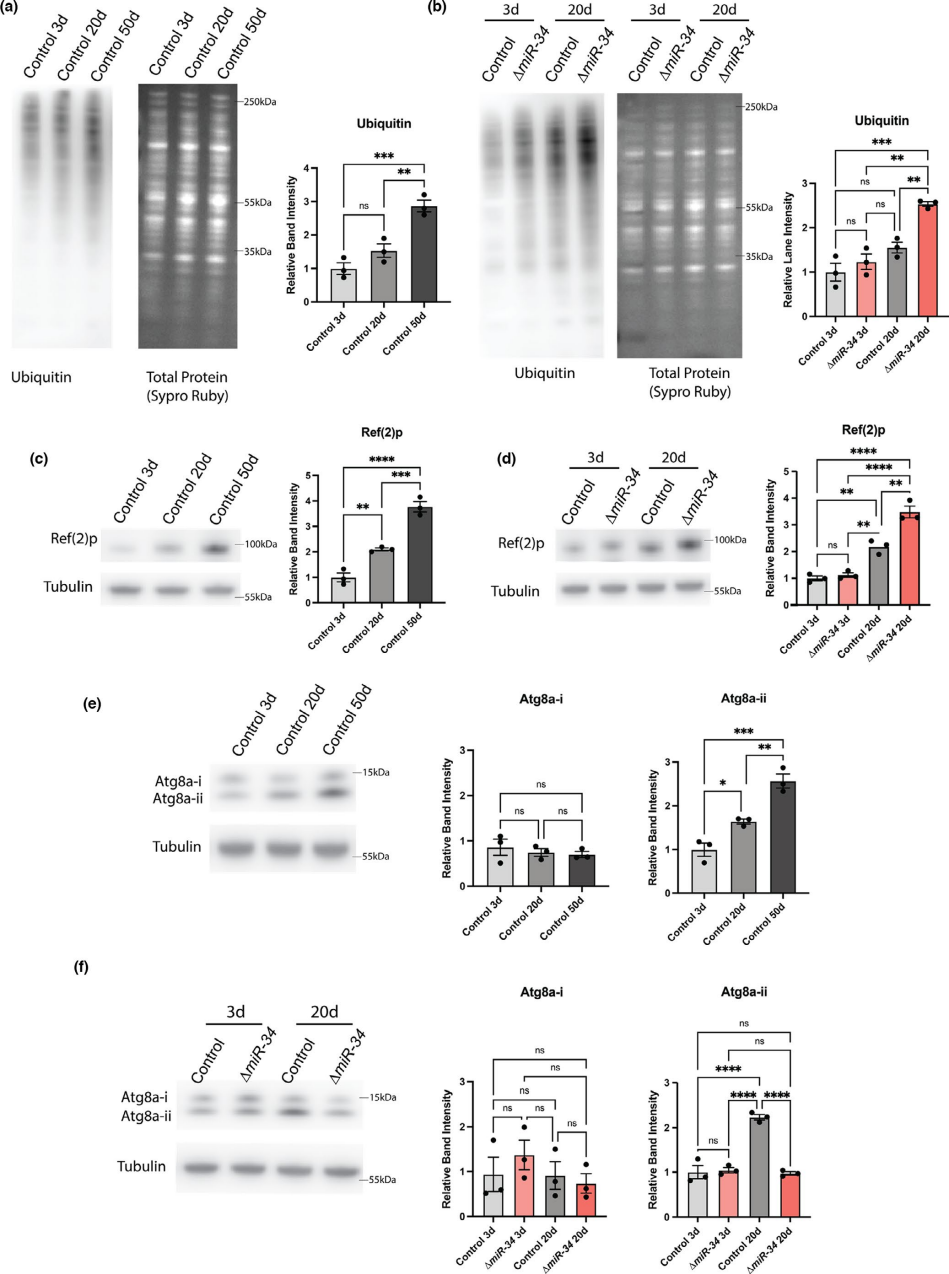
Aged *miR*‐*34* mutant brains have increased levels of markers of protein turnover and autophagy. Western immunoblots with quantitation. For quantitation, *n* = 3 biological replicates; mean ± SEM. (a) Western immunoblot for ubiquitinated protein in the brain with age (3d, 20d, 50d). Ubiquitinated protein significantly increases with age. One‐way ANOVA (*F*(2,6) = 27.0, *p *= 0.001) with Tukey's multiple comparison test. (b) Western immunoblot for ubiquitinated protein in control and *miR*‐*34* mutant brains (3d, 20d). 20d *miR*‐*34* mutants have significantly more ubiquitinated protein. Two‐way ANOVA (Age: *F*(1,8) = 38.86, *p *= 0.0003 | Genotype: *F*(1,8) = 16.68, *p *= 0.0035 | Interaction: *F*(1,8) = 6.3, *p *= 0.036) with Tukey's multiple comparison test. (c) Western immunoblot for Ref(2)p in the brain with age (3d, 20d, 50d). Ref(2)p protein significantly increases with age in the control brain. One‐way ANOVA (*F*(2,6) = 77.96, *p *< 0.0001) with Tukey's multiple comparison test. (d) Western immunoblot for Ref(2)p protein in control and *miR*‐*34* mutant brains (3d, 20d). 20d *miR*‐*34* mutants have significantly more Ref(2)p. Two‐way ANOVA (Age: *F*(1,8) = 147.1, *p *< 0.0001 | Genotype: *F*(1,8) = 23.73, *p *= 0.0012 | Interaction *F*(1,8) = 16.32, *p *= 0.0037) with Tukey's multiple comparison test. (e) Western immunoblot for Atg(8)a levels in the brain with age (3d, 20d, 50d). Processed Atg8a significantly increases with age in the control brain. One‐way ANOVA (Atg8a‐i: *F*(2,6) = 0.46, *p *= 0.6488 | Atg8a‐ii: *F*(2,6) = 36.4, *p *= 0.0004) with Tukey's multiple comparison test. (f) Western immunoblot for Atg8a levels in control and *miR*‐*34* mutant brains (3d, 20d). 20d *miR*‐*34* mutant brains have significantly less processed Atg8a protein than age‐matched control. Two‐way ANOVA (Atg8a‐i – Age: *F*(1,8) = 1.097, *p *= 0.3255 | Genotype: *F*(1,8)=0.1690, *p *= 0.6918 | Interaction: *F*(1,8) = 0.9400, *p *= 0.3607) (Atg8a‐ii – Age: *F*(1,8) = 42.86, *p *= 0.0002 | Genotype: *F*(47.11, *p *= 0.0001 | Interaction: *F*(1,8) = 54.09, <0.0001) with Tukey's multiple comparison test. Significance: *p < 0.05, **p < 0.01, ***p < 0.001, ****p < 0.0001

Translation is a critical aspect of protein homeostasis (referred to as proteostasis, (Vonk et al., 2020)), but proteostasis is also regulated by additional protein processing pathways. We probed the RNA‐sequencing dataset and found evidence of altered activity in key additional proteostasis pathways, including protein folding and turnover (Figure S1f, g). However, given the bidirectional changes in proteostasis‐associated genes, it was difficult to conclude how these transcriptional changes would impact global protein levels. Thus, to define the functional implications, we assayed additional biological features associated with protein accumulation. Excess ubiquitinated protein is a hallmark of impaired protein turnover. With age, the fly brain shows an increase in ubiquitinated protein (Figure 2a) (Nezis et al., 2008). We examined levels of total ubiquitinated protein in young (3d) and aged (20d) control and *miR*‐*34* mutant brains. Young brains had comparable levels of ubiquitinated protein, regardless of genotype. By contrast, the aged *miR*‐*34* mutant brain showed a 1.63‐fold increase in ubiquitinated protein compared to age‐matched controls (Figure 2b). Two‐way ANOVA showed a significant interaction (age‐genotype interaction *p *= 0.0364) between age and genotype, suggesting that loss of *miR*‐*34* impedes ubiquitinated protein turnover with age.

In flies, the *Drosophila* p62 protein, Ref(2)p, accumulates in the brain with age and associates with ubiquitinated protein (Bartlett et al., 2011). The Ref(2)p protein is a selective marker for macroautophagy activity (referred to here as autophagy) (Bartlett et al., 2011; Clausen et al., 2010; Nezis et al., 2008). As previously reported (Bartlett et al., 2011), control animals showed increased levels of Ref(2)p in the brain at 20d and 50d, with 1.8‐fold more total Ref(2)p at 50d compared to 20d (Figure 2c). To determine if this increase was accelerated in the *miR*‐*34* mutant, brains were collected and Ref(2)p levels were measured at 3d and 20d. *MiR*‐*34* mutant brains were similar to controls at 3d, but displayed a statistically significant increase in the levels of Ref(2)p protein at the 20d timepoint (1.59‐fold greater than 20d control brains, age‐genotype interaction *p *= 0.0037) (Figure 2d). This accumulation of Ref(2)p suggests that autophagy is altered in the *miR*‐*34* mutant brain.

Changes in Ref(2)p levels have been associated with changes in autophagosome activity—mutants for the autophagy protein Atg8a have a buildup of Ref(2)p protein (Bartlett et al., 2011). Atg8a (*Drosophila* LC3), a protein necessary for the formation and size of the autophagosome (Xie et al., 2008), is present in two forms: pre‐processed (Atg8a‐i) and processed (Atg8a‐ii). The processed form can be used as a readout for autophagosome formation (Lőrincz et al., 2017). We found that processed Atg8a levels progressively increased normally in the brain with age (Figure 2e). Surprisingly, this increase in Atg8a‐ii levels was not detectable in whole heads, but was prominent in dissected brains. In heads, Atg8a‐i levels were reduced in controls at 20d compared to 3d, consistent with previous reports in heads of Atg8a levels (Simonsen et al., 2008). Unlike Atg8a, both ubiquitinated protein levels and Ref(2)p showed consistent changes between the brain and head (Figure S3a–c). These data suggest that retinal tissue (and outer optic tissue as the lamina is not present in brain samples) in head samples masks changes in autophagy with age that are brain‐specific.

When we examined Atg8a levels in mutant brains, we found that the 3d *miR*‐*34* mutant brain tissue presented similar Atg8a‐ii levels as 3d control brain (Figure 2f). However, at 20d, *miR*‐*34* mutant brains displayed statistically significantly reduced levels of processed Atg8a‐ii relative to age‐matched controls (age‐genotype interaction *p *< 0.0001), with levels comparable to the 3d brain (Figure 2f). This suggests autophagy activity may be altered in the aged *miR*‐*34* mutant brain relative to aged control brains. *MiR*‐*34* mutant animals were also starvation sensitive, a phenotype seen in animals with altered autophagy (Suzuki et al., 2011), although neuron‐specific knockdown of *miR*‐*34* did not show this sensitivity, suggesting the phenotype may be driven by *miR*‐*34* loss in additional tissues (Figure S3d). Together, these findings expand on the result that the loss of *miR*‐*34* drives changes in proteostasis, defining gains in protein accumulation and altered autophagy activity. These data also indicated that *miR*‐*34* may regulate target genes that influence proteostasis in the brain.

### 2.3 Increased H3K27me3 enhances SCA3 PolyQ neurotoxicity and partially mimics the loss in proteostasis seen in the *miR*‐*34* mutant

**FIGURE 3:**
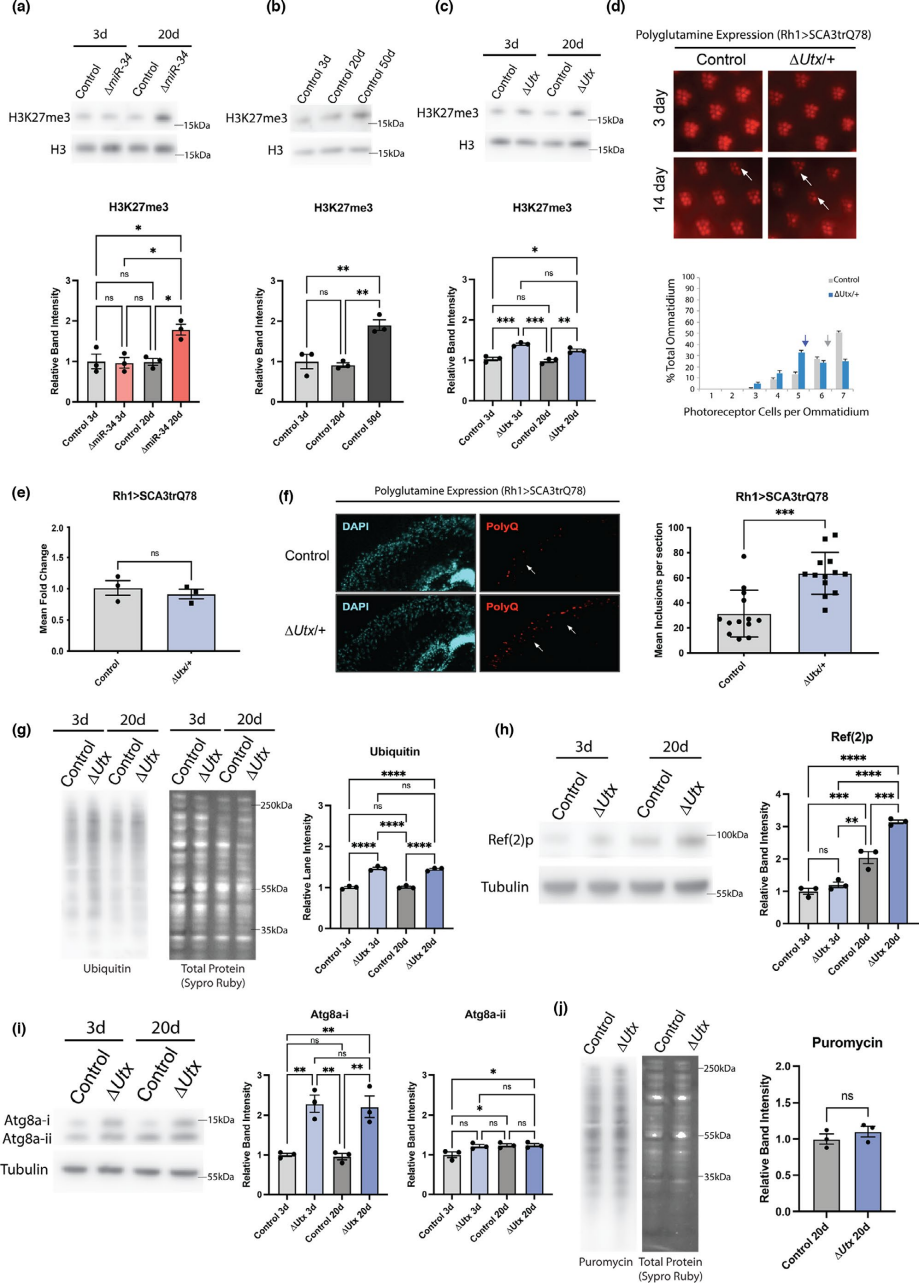
*Utx* reduction increases markers for protein aggregation and enhances SCA3 neurodegeneration. (a) Western immunoblot for H3K27me3 in control and *miR*‐*34* mutant brains (3d, 20d). (*n* = 3 biological replicates, mean ± SEM, two‐way ANOVA (Age: *F*(1,8) = 8.741, *p*=0.0182 | Genotype: *F*(1,8) = 7.761, *p *= 0.0237 | Interaction: *F*(1,8) = 9.253, *p *= 0.016) with Tukey's multiple comparison test). (b) Western immunoblot for H3K27me3 in the brain with age (3d, 20d, 50d). (*n* = 3 biological replicates, mean ± SEM, one‐way ANOVA (*F*(2,6) = 17.12, *p *= 0.0033) with Tukey's multiple comparison test). (c) Western immunoblot for H3K27m3 in control and *Utx* mutant brains (3d, 20d). (*n* = 3 biological replicates, mean ± SEM, two‐way ANOVA (Age: *F*(1,8) = 7.484, *p *= 0.0256 | Genotype: *F*(1,8) = 71.93, *p *< 0.0001 | Interaction: *F*(1,8) = 2.129, *p *= 0.1827) with Tukey's multiple comparison test). (d) Pseudopupil assay presenting changes in photoreceptor degeneration of SCA3trQ78‐expressing animals in control and *Utx*/+ heterozygote backgrounds (*n* = 150 ommatidia/condition). Histogram showing the distribution of photoreceptors per ommatidium at 14d (mean noted with arrow). (e) qPCR for SCA3trQ78 transcript abundance in control and *Utx*/+ heterozygote backgrounds (1d). (*n* = 3 biological replicates, mean ± SEM, Student's *t* test). (f) Cryosections on heads expressing SCA3trQ78 in control and *Utx*/+ heterozygote backgrounds (*n* = 13 flies/condition, mean ±SEM, Student's *t* test) (1d). (g) Western immunoblot for ubiquitinated protein in 3d and 20d control and *Utx* mutant brains. (*n* = 3 biological replicates, mean ± SEM, two‐way ANOVA (Age: *F*(1,8) = 0.03235, *p *= 0.8617, Genotype: *F*(1,8) = 355.8, *p *< 0.0001, Interaction: *F*(1,8) = 0.4783, *p *= 0.5088) with Tukey's multiple comparison test). (h) Western immunoblot for Ref(2)p protein in 3d and 20d control and *Utx* mutant brains. 20d (*n* = 3 biological replicates, mean ± SEM, two‐way ANOVA (Age: *F*(1,8) = 169.2, *p *< 0.0001 | Genotype: *F*(1,8) = 32.19, *p *= 0.0005 | Interaction: *F*(1,8) = 15.44, *p *= 0.0044) with Tukey's multiple comparison test). (i) Western immunoblot for Atg(8)a protein in 3d and 20d control and *Utx* mutant brains. (n=3 biological replicates, mean ± SEM, two‐way ANOVA (Atg8a‐i – Age: *F*(1,8) = 0.1192, *p *= 0.7388 | Genotype: *F*(1,8) = 52.62, *p *< 0.0001 | Interaction: *F*(1,8) = 0.01055, *p* = 0.9207) (Atg8a‐ii – Age: *F*(1,8) = 6.622, *p *= 0.0330 | Genotype: *F*(1,8) = 4.882, *p *= 0.0581 | Interaction: *F*(1,8) = 4.499, *p *= 0.0667) with Tukey's multiple comparison test). *Utx* mutants had significantly more Atg8a‐i, but there was no significant difference in processed Atg8a‐ii protein. (j) Western immunoblots of puromycin‐labeled proteins in 20d control and *Utx* mutant brains. (*n* = 3 biological replicates, mean ± SEM, Student's *t* test). Significance: *p < 0.05, **p < 0.01, ***p < 0.001, ****p < 0.0001

Only one target pathway of *miR*‐*34* has been implicated in protein aggregation to date. Mutations in *miR*‐*34* targets *Pcl* and *Su(z)12* (subunits of the histone methyltransferase PRC2) have been shown to suppress protein aggregation of the pathogenic SCA3trQ78 protein and prevent neurotoxicity—this suppression correlates with reduced H3K27me3. The *miR*‐*34* mutant brain shows increased H3K27me3 with age, achieved at least in part through upregulation of these components of PRC2 (Figure 3a) (Kennerdell et al., 2018). H3K27me3 levels normally also increase with age in flies (Figure 3b) and mice and correlate with increased protein aggregation (Booth & Brunet, 2016). We hypothesized that the early accumulation of H3K27me3 in *miR*‐*34* mutants may drive the dysregulation of proteostasis and susceptibility to neurodegeneration that is seen in the aged brain. To determine if increasing H3K27me3 levels alone could mimic the proteostasis dysregulation seen in the *miR*‐*34* mutant, we examined these pathways in a mutant for the sole *Drosophila* H3K27me3 histone demethylase *Utx* (Smith et al., 2008). *Drosophila* mutants for *Utx* show increased H3K27me3 levels in the brain at both 3d and 20d (Figure 3c) and have a reduced lifespan (Ma et al., 2018).

We first assessed the impact of the *Utx* mutation on neurodegeneration. *Utx* mutants or controls were outcrossed to flies expressing the pathogenic SCA3trQ78 protein and neurodegeneration was assessed within the photoreceptor cells with age. Normally, flies have 7 ± 0 (mean, ±SEM) photoreceptors per ommatidial unit, which are fully maintained with age (Bilen & Bonini, 2007). When the pathogenic SCA3 protein is expressed, it induces photoreceptor degeneration with age. At 3d, all photoreceptors were present in both control and *Utx*/+ animals. At 14d, animals expressing SCA3trQ78 now had 6.18 ± 0.04 photoreceptors/ommatidial unit, whereas animals with reduced *Utx* showed fewer photoreceptors/ommatidial unit (5.49 ± 0.05 (Figure 3d)). In tandem, we confirmed by real‐time qPCR that reduction of *Utx* did not impact transcription of the Sca3trQ78 transgene (Figure 3e). These data indicate that increasing H3K27me3 through reduced *Utx* gene function enhances polyglutamine neurotoxicity.

We confirmed that the increased polyglutamine neurotoxicity was coupled with increased aggregation of the SCA3trQ78 protein by assessing polyglutamine protein in cryosections of fly heads. 1d after SCA3trQ78 expression in photoreceptors, control animals had an average of 29 ± 19 inclusions per section, whereas *Utx*/+ animals had 63 ± 16 inclusions (Figure 3f). The increase in SCA3trQ78 inclusions suggests that reduction of *Utx* may enhance neurodegeneration through impaired protein regulation.

Since animals with reduced *Utx* activity enhance neurodegeneration like *miR*‐*34* mutants, we assessed if proteostasis was altered in a similar fashion. We first examined levels of ubiquitinated protein in the brain by western immunoblot. As with *miR*‐*34* mutants, at 20d there was a significant increase (1.57‐fold gain, *p *< 0.0001) in ubiquitinated protein in *Utx* mutant brain tissue compared to age‐matched control animals (Figure 3g). We also saw an increase in ubiquitination in the 3d *Utx* mutant brain (1.46‐fold gain, *p *< 0.0001), correlating with the significant increase in H3K27me3 levels already present in the 3d *Utx* mutant brain. Therefore, reduction of *Utx* alone is capable to drive gains in ubiquitinated protein, independent of brain age. *Utx* mutants additionally had a 1.54‐fold increase in Ref(2)p in the 20d brain (age‐genotype interaction *p *= 0.0044) (Figure 3h). These results indicate that the loss of H3K27me3 histone demethylase *Utx* increases protein ubiquitination and Ref(2)p buildup in the adult brain.

We then examined autophagosome formation and translation activity. Western immunoblots were performed on 3d and 20d control and *Utx* mutant brains to assess pre‐processed and processed Atg8a. *Utx* mutants showed an age‐independent increase in Atg8a‐i levels relative to control brains: both 3d and 20d *Utx* mutants also showed similar levels of processed Atg8a‐ii compared to the 20d control brain, in contrast to *miR*‐*34* mutants (Figure 3i). We then assessed protein translation with the puromycin feeding assay. Brains from 3d and 20d control and *Utx* mutants displayed no differences in levels of puromycin‐tagged protein (Figure 3j, Figure S4b,c). These findings indicate that, whereas the loss of the H3K27me3 demethylase *Utx* dysregulates proteostasis, *Utx* reduction mimics only a select set of the biological effects characteristic of the *miR*‐*34* mutant. These findings raised the possibility that additional targets of *miR*‐*34*, beyond the regulation of H3K27me3 through PRC2, are involved in altered translation and autophagy activity in the brain with age.

### 2.4 *miR‐34* is predicted to target *Lst8*, a subunit of TORC1

**FIGURE 4:**
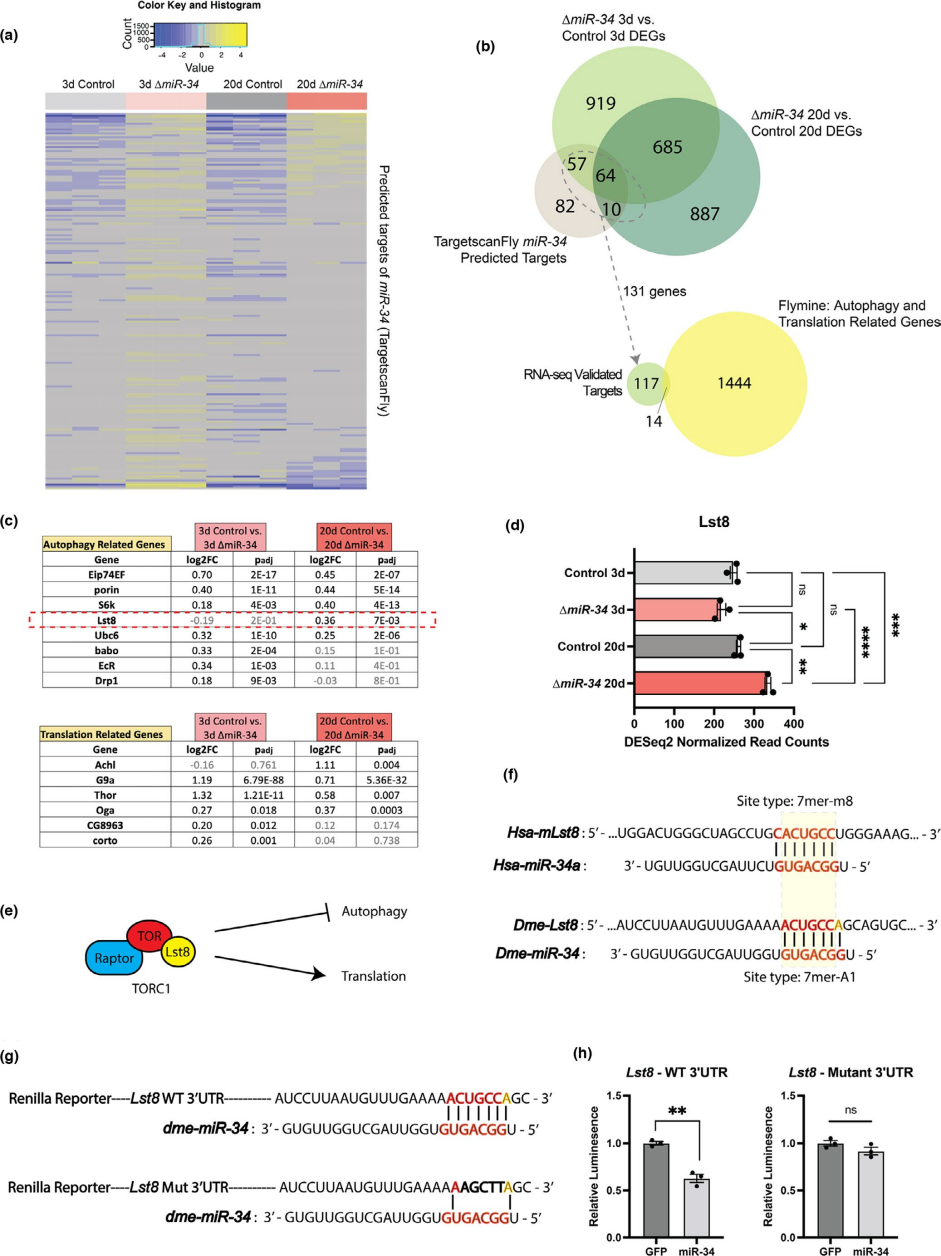
*miR*‐*34* is predicted to target TORC1 subunit, *Lst8*. (a) Heat map comparing transcript levels for all TargetscanFly predicted targets of *Drosophila miR*‐*34* (b) Venn Diagram (top) presenting the overlap between genes significantly upregulated in the 3d (*miR*‐*34* vs. control) and 20d (*miR*‐*34* vs. control) brain vs. predicted targets of *Drosophila miR*‐*34*. Venn Diagram (bottom) presenting overlap between RNA‐seq verified targets of *miR*‐*34* and Translation‐related and Autophagy‐related gene lists derived from Flymine database. (c) RNA‐seq validated targets of *miR*‐*34* associated with autophagy and translation based on curated gene lists from the Flymine database. (d) RNA‐seq normalized reads for *Lst8*. *Lst8* is significantly upregulated in the 20d *miR*‐*34* mutant brain (*n* = 3 biological replicates, mean ±SEM, two‐way ANOVA (Age: *F*(1,8) = 60.88, *p *< 0.0001 | Genotype: *F*(1,8) = 6.968, *p *= 0.0297 | Interaction: *F*(1,8) = 40.04, *p *0.0002) with Tukey's multiple comparison test). (e) Schematic showing the role of TORC1 in regulating both translation and autophagy. (f) 3’UTR *Lst8* and *mLst8* seed sequences for *miR*‐*34*. (g) Wild‐type and mutant seed sequences for *Lst8* 3’UTR reporter constructs. (h) Luciferase reporter assay for WT and *miR*‐*34* mutant 3’UTR with co‐expression of *miR*‐*34* or GFP control. Upregulation of *miR*‐*34* significantly reduced the activity of Renilla luciferase fused to only the WT 3’UTR of *Lst8* (n=3 wells/condition, mean ± SEM, Student's *t* test). Significance: *p < 0.05, **p < 0.01, ***p < 0.001, ****p < 0.0001

To identify additional targets modulated by *miR*‐*34* in the brain, we cross‐referenced the list of 213 computationally identified *miR*‐*34* targets (Agarwal, 2018) against the *miR*‐*34* brain RNA‐seq dataset (Figure 4a). 131 of the predicted targets were significantly upregulated in the mutant brain, consistent with how *miR*‐*34* may regulate its targets (Figure 4b). Select targets were uniquely upregulated: 51 targets were uniquely upregulated at 3d, and 3 were uniquely upregulated at 20d. Altogether, these 131 targets comprise an RNA‐seq validated *miR*‐*34*‐target list of brain genes.

Next, we determined whether any of these targets were associated biologically with translation or autophagy. We defined a list of all translation‐related and autophagy‐related genes using the Flymine database (File S4). We compared the 131 transcriptionally upregulated *miR*‐*34* target genes with this list to explore if any of the RNA‐seq validated targets were implicated in pathways of translation or autophagy.

This approach highlighted 14 *miR*‐*34* targets with association with translation or autophagy, although no predicted targets were core components of either pathway (Figure 4b, c). *Lst8* was identified as an intriguing candidate (Figure 4c, d). As a subunit of TOR Complex 1 (TORC1), Lst8 binds the kinase domain of TOR and stimulates TORC1 activity (Kamada et al., 2010; Loewith & Hall, 2011; Miron & Sonenberg, 2001; Noda, 2017; Rabanal‐Ruiz et al., 2017)—increased TORC1 activity has been shown to stimulate translation initiation and inhibit autophagosome formation (Figure 4e) (Aylett et al., 2016; Couso et al., 2020).

We identified a putative *miR*‐*34* target sequence in the 3’ UTR of *Lst8* (Figure 4f) that matched the canonical 7mer‐A1 microRNA‐binding pattern (Agarwal et al., 2015). Of note, the mammalian homolog of *Lst8*, *mLst8*, also has a 3’ UTR microRNA seed sequence for the *miR*‐*34* microRNA family (Figure 4f), suggesting *Lst8* may be an evolutionarily conserved target. We next utilized *Drosophila* S2R+ cells to determine whether *miR*‐*34* could regulate the 3’ UTR of *Lst8*. We designed a plasmid reporter bearing either the wild‐type (WT) 3’ UTR of *Lst8* with the *miR*‐*34* target sequence, or a 3’ UTR of *Lst8* with a mutated *miR*‐*34* target sequence (Figure 4g). The plasmids were co‐transfected with a plasmid expressing either *miR*‐*34* or GFP control, as well as a plasmid for transfection efficiency. *miR*‐*34* upregulation reduced relative Renilla activity with the wild‐type *Lst8* 3’ UTR, but had no effect on the mutant construct (Figure 4h). These data indicate that *miR*‐*34* can regulate the 3’ UTR of *Lst8*.

We tested if upregulation of *Lst8* was sufficient to drive changes in translation and autophagy as seen in the aged *miR*‐*34* mutant brain. Upregulation of *Lst8* did not lead to changes in either global translation activity or Atg8a levels in 20d brains (Figure S4c, d). Considering the nature of microRNA regulation, we propose that alternative targets of *miR*‐*34*, such as other members of the predicted translation‐autophagy *miR‐34* targets (Figure 4d) and the established targets within the H3K27me3 regulation pathway (Figure S3a), are contributors to the mutant phenotype. Together, these results define a new target of *miR‐34* in *Lst8* and suggest that the global proteostasis changes seen in the

**FIGURE 5:**
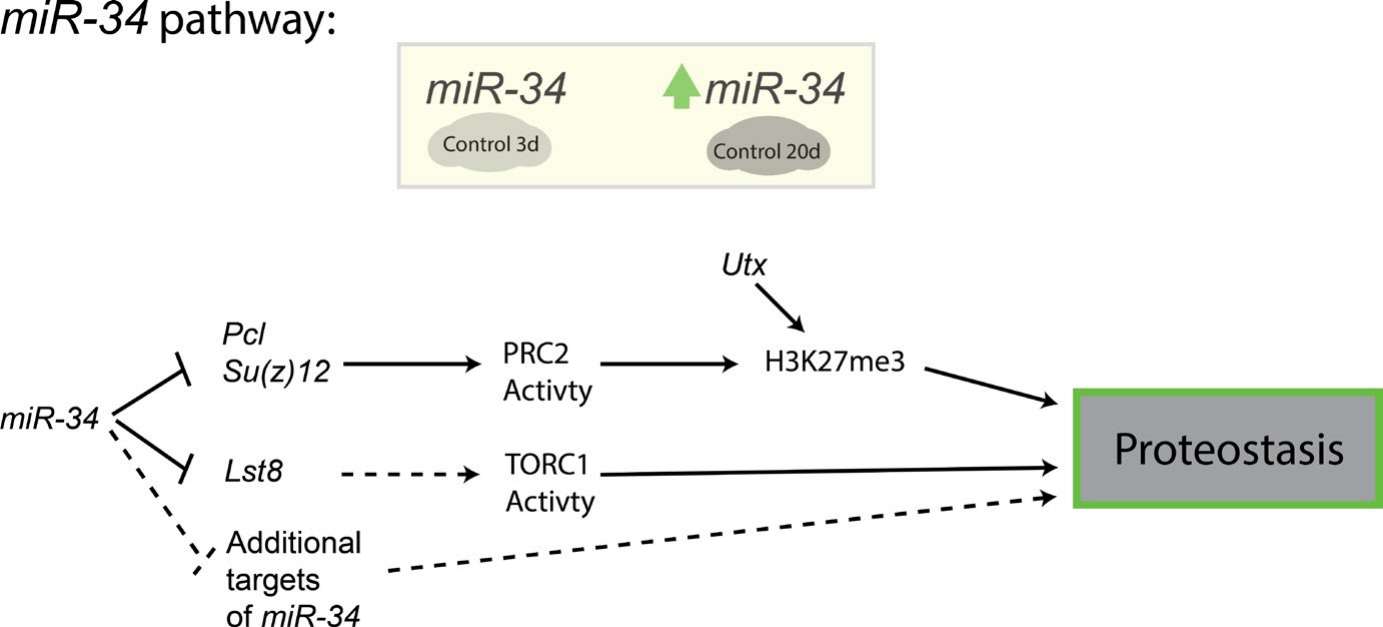
Role of *miR*‐*34* in maintaining brain proteostasis with age. Schematic presenting the role of *miR*‐*34* in maintaining proteostasis in the *Drosophila* brain

*miR*‐*34* mutant brain are a summation of multiple misregulated targets (Figure 5).

## 3 DISCUSSION

We provide new insight into the importance of the highly conserved microRNA *miR*‐*34* in regulating proteostasis and maintaining healthy brain aging. We find that the loss of *miR*‐*34* drives transcriptional changes in both the 3d and 20d brain. The transcriptional changes between the 20d mutant and control brain show significant overlap with transcriptional changes seen with advanced age (50d control), consistent with prior observations that loss of *miR*‐*34* promotes aging gene changes in the brain coupled with advanced neurodegeneration. These data develop our understanding of cellular processes regulated by *miR*‐*34* in the brain and provide a new understanding into the loss of *miR*‐*34* as a modifier of aging and age‐associated impacts to the brain.

By focusing attention on proteostasis, we identified that aged *miR*‐*34* mutant brains have a statistically significant increase in the accumulation of ubiquitinated protein and p62/Ref(2)p in the 20d brain relative to their age‐matched controls. Accumulation of both proteins with age has been observed in the nervous system of flies, mice, and humans and has been associated with neuronal loss and susceptibility to neurodegenerative disease (Bartlett et al., 2011; Schmidt et al., 2021; Simonsen et al., 2008).

Using levels of Atg8a as a marker of autophagosome formation, control animals showed a brain‐specific increase in processed Atg8a with age (see Figure 1e, Figure S1). This contrasts with reports measuring Atg8a levels in fly heads, suggesting that retinal tissue masks Atg8a changes in the brain. Aged *miR*‐*34* mutant brains, on the other hand, had reduced processed Atg8a protein compared to control brains. The reduction in Atg8a processing in the aged *miR*‐*34* mutant suggests that autophagy activity is altered with the loss of *miR*‐*34*, although it is unclear if there is an increase or decrease in autophagic flux.

Selectively increasing H3K27me3 via loss of the histone demethylase *Utx* phenocopied only select proteostasis impairments observed in the *miR*‐*34* mutant brain. These findings suggested that dysregulation of additional targets of *miR*‐*34* may underlie aspects of altered proteostasis in the mutant brain. We showed that *miR*‐*34* can target *Lst8*, a subunit of TORC1, however, upregulation of *Lst8* alone did not recapitulate the global changes in proteostasis seen in the *miR*‐*34* mutant brain.

Additional targets of *miR*‐*34* presumably contribute to the changes in proteostasis (see Figure 4a, b, File S5). We also recognize that select predicted targets of *miR*‐*34*, when upregulated, may not contribute to the observed *miR*‐*34* mutant phenotypes, and instead may drive alternative responses. Moreover, many targets may have modest although important changes in the *miR*‐*34* mutant brain; misregulation of multiple targets presumably contributes to the overall biological outcome. Further detailed study of additional targets and understanding of how these gene target functions intersect in *miR*‐*34* and the normal brain with age should reveal pathways that are normally regulated in the brain with age, as well as new pathways whose activity could be adjusted to promote healthy brain aging.

In conclusion, here we have identified a novel relationship between *Drosophila miR*‐*34* and regulation of proteostasis in the brain (Figure 5). Two *miR*‐*34* pathways may contribute to aspects of the proteostasis impairment in *miR*‐*34* mutants: an increase of H3K27me3 (achieved here via reduction in *Utx*) is sufficient to drive protein aggregation; however, as *Utx* reduction does not fully recapitulate *miR*‐*34* mutant effects, we posit that a conserved novel target, *Lst8*, may also contribute to altered proteostasis through its activity with TORC1. Lastly, we identify additional predicted targets of *miR*‐*34* that may regulate brain proteostasis with age. Together, these findings indicate that the *miR*‐*34* axis defines critical gene functions that can regulate proteostasis in the aging fly brain. Given conservation of *miR*‐*34*, these gene functions may also be of impact for the mammalian brain.

## 4 MATERIALS AND METHODS

### 4.1 Fly stocks

All crosses were performed at 26ºC on standard molasses fly food. Males were used for all experiments. Flies were transferred to fresh food every 1‐2d. For *Lst8* upregulation experiments, food was prepared by adding 50 μl of 4mg/ml of RU468 dissolved in 100% EtOH or by adding 50 μl of 100% EtOH. DaGS>Lst8 flies were put on either +RU486 or +Vehicle food after eclosion.

### 4.2 Western Immunoblots (WB)

For fly WB, 10 brains (or heads) were dissected for each replicate and homogenized in 50 μl of 1X NuPAGE LDS sample buffer. WB were performed using an Invitrogen XCell SureLock blot system with a 4–12% Bis‐Tris gel (5 μl sample/lane) and transferred overnight to a PVDF membrane (Wet transfer, 1x Dunn Carbonate Transfer Buffer). Following transfer, membranes were blocked in 5% Milk/TBST for 1 hr. Blots were probed overnight for primary antibodies, washed 3X for 10 min in TBST, and probed for 1 h in secondary antibody in 5% Milk/TBST. Blots were then washed 3X for 5 min and imaged using Amersham ECL Prime Detection Reagent and Amersham Imager 600. All quantifications are based on an average from three independent lanes/condition (each lane is a biological replicate).

### 4.3 Lifespan assay

Flies were transferred to fresh food and scored every 2d. 120 flies were used for each condition.

### 4.4 Starvation assay

Flies were placed in vials with water‐soaked 3 mm filter paper (Thermo Fischer Scientific; Waltham, MA, USA). Flies were transferred to fresh water‐soaked filter paper every 12 h and scored. 120 flies were used for each condition.

### 4.5 Puromycin feeding assay

Flies were placed on 600 μM puromycin P8833 (Sigma‐Aldrich; Darmstadt, Germany)/2% agar/5% sucrose food. After 24 h, fly brains (10 brains/condition) were dissected for western immunoblots.

### 4.6 Feeding measurement assay

Flies were fed standard food mixed with 1% FD&C Blue #1 (SPS Alfachem, Lexington, MA) for 24 h. After 24 h, whole flies (5 flies/replicate) were collected and homogenized in 1% Triton‐X in PBS to measure blue dye incorporation. Three independent replicates were performed for each condition. Blue dye absorbance (630 nm) was measured with a Nanodrop.

### 4.7 RNA Sequencing

Twenty brains were used for each replicate (three biological replicates/condition). Brains were dissected and put into Trizol (Thermo Fischer Scientific). RNA was isolated using a standard Trizol protocol (Goodman et al., 2019). RNA was purified using RNA Clean & Concentrator Kit R10114 (Zymo Research, Irvine, CA). RNA sequencing by Admera Health BioPharma Services (South Plainfield, NJ), using TruSeq Stranded mRNA Kit with PolyA selection (Illumina; San Diego, CA).

### 4.8 RNA‐Sequencing analysis

Reads were mapped to the *Drosophila* Genome r6.36 using HISAT2 default parameters (Kim et al., 2019). HTSeq counts python package was used to generate gene counts against FlyBase version6.63 GTF file (Anders et al., 2015). Differential gene analysis was performed using DESeq2 package (Love et al., 2014).

### 4.9 Flymine dataset mapping

Gene ontology analysis was performed using Flymine Database (Lyne et al., 2007). Significantly upregulated and downregulated genes for 3d control vs. *miR*‐*34* mutant and 20d control vs. *miR*‐*34* mutant against a background list of all expressed genes (15,114) were analyzed. Significance was determined by a Benjamini–Hochberg *p*_adj _< 0.05 cutoff. Gene lists for proteostasis network were curated by searching each pathway term on Flymine v.51, filtering for organism (*Drosophila melanogaster*) and then category (Gene).

### 4.10 Targetscan dataset mapping

Predicted *Drosophila miR*‐*34* targets (*miR*‐*34*‐5p) were taken from TargetscanFly 7.2 (Agarwal, 2018; Agarwal et al., 2015) for mapping against all differentially expressed genes for 3d control vs. *miR*‐*34* and 20d control vs. *miR*‐*34*.

### 4.11 Pseudopupil assay

Pseudopupil assay was performed as described (Kennerdell et al., 2018). Briefly, flies were anesthetized using CO_2_ and heads were removed for imaging. Heads were placed on a slide and positioned with petroleum jelly. The light path was posterior along the axis of the ommatidia. Heads were imaged with bright field imaging using a 100x oil immersion objective on Leica DM6000B microscope. Ten flies were used for each condition, with a total of 150 ommatidia quantified for each condition.

### 4.12 Quantitative RT‐PCR

RNA was isolated from brains (15 brains/replicate, three biological replicates/condition) using standard Trizol protocol. 200–300 ng of RNA was used to generate cDNA with High Capacity cDNA Reverse Transcription Kit (Thermo Fischer Scientific). qPCR reactions were set up using SYBR Green Fast Reagent (Thermo Fischer Scientific) and analyzed using Applied Biosystems ViiA 7 Real‐Time PCR System. Mean fold change was determined using ΔΔ*C*_t_ method.

### 4.13 Cryosection immunohistochemistry

Cryosections were performed as described (Kennerdell et al., 2018) with minor alterations. Heads were blocked in OCT medium, and 12 μM sections were cut on a CM3050S cryostat (Leica, Buffalo Grove, IL). Sections were fixed in 0.5% paraformaldehyde in phosphate‐buffered saline (PBS) for 10 min. Slides were blocked for 1 h in PBSG (PBS, 0.5% Triton, 1% goat serum) followed by overnight incubation in primary antibody rat anti‐HA (Roche 3F10, 1:100). Goat anti‐rat 549 (Invitrogen A11007, 1:200) was used as a secondary. Sections were stained with Hoechst 33342 for 10 min and imaged using Leica DM6000B microscope. Thirteen heads were quantified for each condition.

### 4.14 Luciferase assay

Reporter constructs for the *Lst8* WT 3’ UTR and Mut 3’UTR were cloned into pMT‐Renilla plasmid at the BamHI/SalI sites. Seed sequence mutations were created using the Quik‐change mutagenesis system (Stratagene; La Jolla, CA). Luciferase assays were performed as described (Kennerdell et al., 2018). For each co‐transfection into S2R+ cells (6‐well plate), either pMT‐Renilla‐*Lst8* WT 3’ UTR or pMT‐Renilla‐*Lst8* Mut 3’ UTR was transfected with either pMT‐*miR*‐*34* or pMT‐GFP. A control pMT‐Firefly plasmid was transfected with each condition. Following transfection, cells were transferred to a 96‐well plate. Luciferase assays were performed using the Dual‐Glo Luciferase Assay System (Promega, Madison, WI). 3 wells were quantified for each condition on a Tecan Infinite 200 Pro Plate Reader (Tecan; Männedorf, Switzerland). S2R+ cells were maintained using standard conditions as provided from *Drosophila* Genomics Resource Center.

### 4.15 Statistics

Statistics were performed using Prism (GraphPad Software; La Jolla, CA). Data were assumed to be normally distributed. No statistical method was used to predetermine sample size; numbers used are similar to previously published studies (Goodman et al., 2019; Kennerdell et al., 2018; Liu et al., 2012).

## CONFLICTS OF INTEREST

None declared.

## AUTHOR CONTRIBUTIONS

A.R.S and N.M.B designed research. A.R.S performed experiments and analyzed data. T.T.T helped analyze RNA‐seq dataset and provided technical support on wet laboratory experiments. A.R.S and N.M.B wrote the manuscript.

## Supporting information

Supplementary Material

## Data Availability

The datasets generated during the current study are available under accession GSE184559 in NCBI GEO and upon request.
