# Supplementary material for: Loss of miR‐34 in Drosophila dysregulates protein translation and protein turnover in the aging brain
Source: Aging Cell. 2022 Feb 15;21(3):e13559. doi: 10.1111/acel.13559 (PMC8920459; doi:10.1111/acel.13559)
Supplement: Supplementary file 1 — Supplementary Material [file ACEL-21-e13559-s001.docx]

***Supplemental Material to:***

**Loss of *miR-34* in *Drosophila* dysregulates protein translation and protein turnover in the aging brain**

Ananth R. Srinivasan, Tracy T. Tran, Nancy M. Bonini

Department of Biology

University of Pennsylvania

Philadelphia, PA 19104





**Supplemental Figure 1. Transcriptomic profiling of the *miR-34* mutant brain with age**

**(a)** MA plot showing differential gene expression for 3d *miR-34* vs. 3d control brains. Green dots represent genes upregulated (p_adj_<0.05) and red dots represent genes downregulated (p_adj_<0.05) in the *miR-34* mutant. **(b)** Bar plot presenting significant differentially expressed genes (p_adj_<0.05). **(c)** MA plot showing differential gene expression for 50d control brains vs. 20d control brains. Green dots represent genes upregulated (p_adj_<0.05) and red dots represent genes downregulated (p_adj_<0.05) in the 50d brain. **(d)** Venn diagram showing significant overlap between genes that are significantly upregulated (left) and significantly downregulated (right) in 20d *miR-34* vs control and genes that go up in 50d vs. 20d control. (Upregulation: 5.26-fold over-enriched | Downregulation: 3.28-fold over-enriched). **(e)** Top 5 GO Terms against significantly downregulated genes in the 20d *miR-34* mutant vs 20d control brain **(f)** Heat map showing the DESeq2 normalized read counts for all cytoplasmic translation genes across 3d control, 3d *miR-34*, 20d control, and 20d *miR-34* **(g)** Schematic for the proteostasis network and volcano plots presenting significant gene changes in key proteostasis pathways between the 20d *miR-34* brain and age-matched control.





**Supplemental Figure 2. In head tissue, *miR-34* mutants have accumulation of ubiquitinated protein, Ref(2)p, and reduced autophagy**

**(a)** Western immunoblot for ubiquitinated protein in control and *miR-34* mutant heads (3d, 20d). (n=3 biological replicates, mean ± SEM, two-way ANOVA (Age: F(1,8)=23.52, p=0.0013 | Genotype: F(1,8)=17.79, p=0.0029 | Interaction: F(1,8)=5.637, p=0.0449) with Tukey’s multiple comparison test). **(b)** Western immunoblot for Ref(2)p in control and *miR-34* mutant heads (3d, 20d). (n=3 biological replicates, mean ± SEM, two-way ANOVA (Age: F(1,8)=6.850, p=0.0308 | Genotype: F(1,8)=19.27, p=0.0023 | Interaction: F(1,8)=6.850, p=0.0308) with Tukey’s multiple comparison test). **(c)** Western immunoblot for Atg8a levels in control and *miR-34* mutant heads (3d, 20d). (n=3 biological replicates, mean ± SEM, two-way ANOVA (Atg8a-i – Age: F(1,8)=8.7655, p=0.0181 | Genotype: F(1,8)=2.766, p=0.1348 | Interaction: F(1,8)=9.761, p=0.0141) (Atg8a-ii – Age: F(1,8)=5.096, p=0.0539 | Genotype: F(1,8)=3.423, p=0.1015 | Interaction: F(1,8)=3.785, p=0.0876 ) with Tukey’s multiple comparison test). **(d)** Starvation survival curve comparing 20d control and *miR-34* mutant animals and 20d scramble-sponge (control) and *miR-34*-sponge animals (sponge driven in neurons with elav-GAL4) (n=120 animals/group). **(e)** Dye feeding assays show no significant differences in feeding between 3d and 20d *miR-34* mutants and controls. (n=3 biological replicates, mean ±SEM, Student’s t-test). Significance: * p<0.05, ** p<0.01, *** p<0.001.


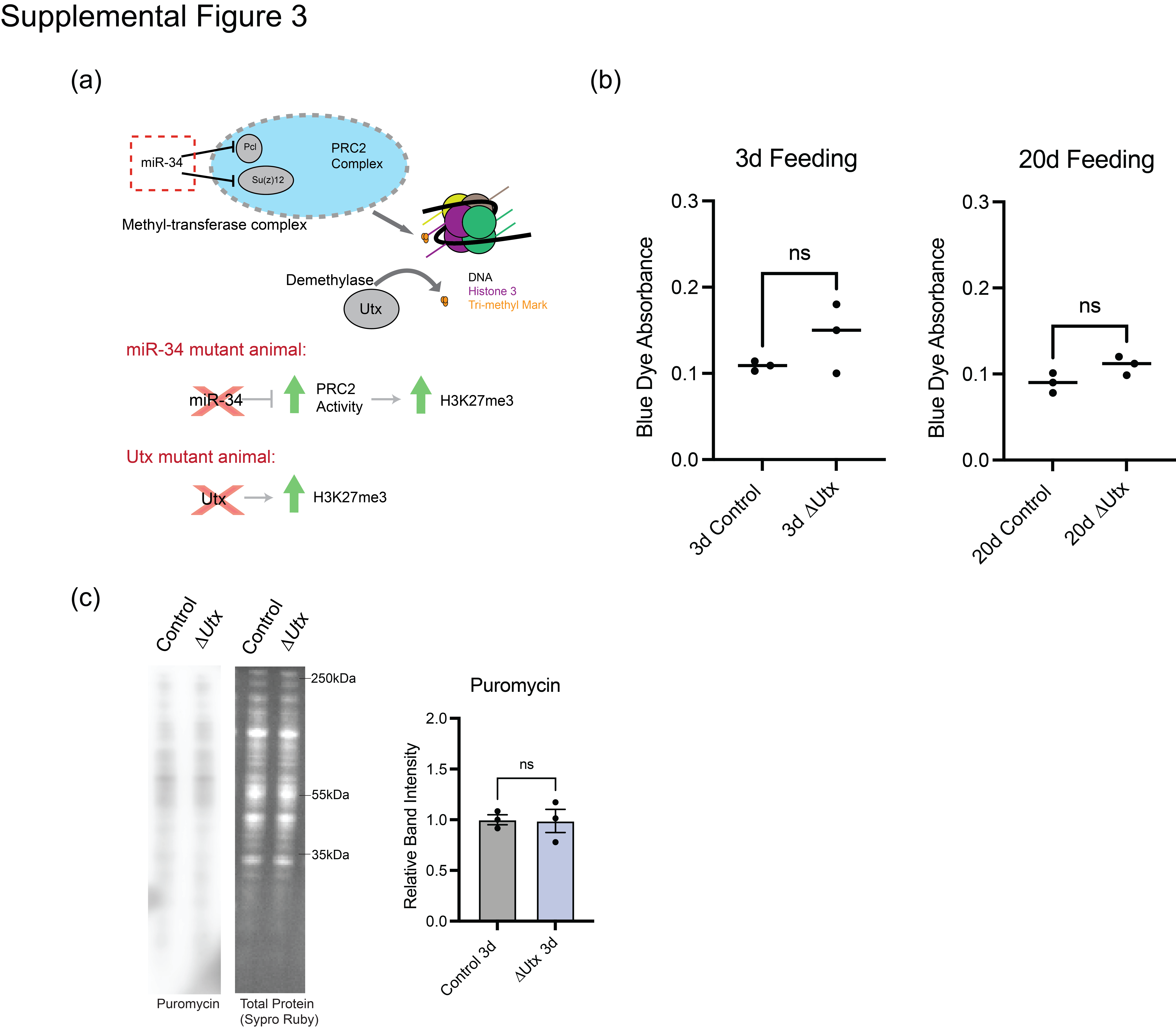


**Supplemental Figure 3. Characterizing the *Utx* mutant in the context of *miR-34* phenotypes**

**(a)** Schematic showing the relationship between *miR-34*, *Utx,* and H3K27me3. **(b)** Dye feeding assays show no significant differences in feeding between 3d and 20d *Utx* mutants and controls. (n=3 biological replicates, mean ±SEM, Student’s t-test). **(c)** Western immunoblots of puromycin-labelled proteins in 3d control and *Utx* mutant brains. *Utx* mutants have no change in puromycin-labeled protein vs. control in the 3d brain. (n=3 biological replicates, mean ±SEM, Student’s t-test).


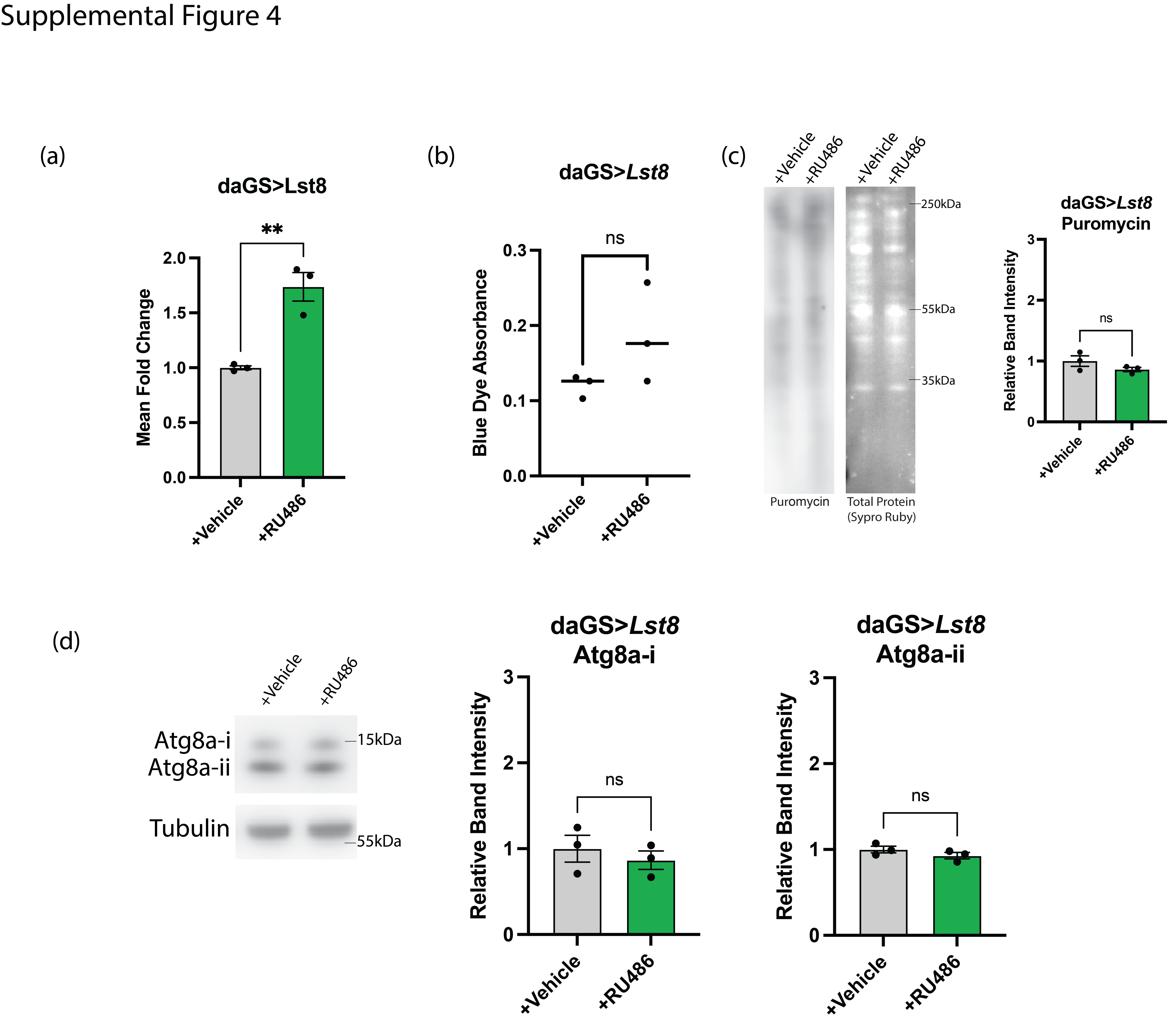


**Supplemental Figure 4. Characterizing the upregulation of *Lst8* and the measurement of phosphorylated Thor in *Drosophila***

**(a)** qPCR quantifying the level of *Lst8* upregulation in 20d daGS>Lst8 fly brains. Flies fed RU486 have significantly more *Lst8* transcript than those on vehicle (n=3 biological replicates, mean ±SEM, Student’s t-test). **(b)** Dye feeding assays show no significant differences in feeding between daGS>Lst8 20d flies on Vehicle or RU486. (n=3 biological replicates, mean ±SEM, Student’s t-test). **(c)** Western immunoblot comparing puromycin-labeled protein in 20d daGS>Lst8 flies fed Vehicle or RU486. (n=3 biological replicates, mean ±SEM, Student’s t-test). **(d)** Western immunoblot comparing Atg(8)a levels in 20d daGS>Lst8 flies fed Vehicle or RU486. (n=3 biological replicates, mean ±SEM, Student’s t-test). Significance: ** p<0.01.

**
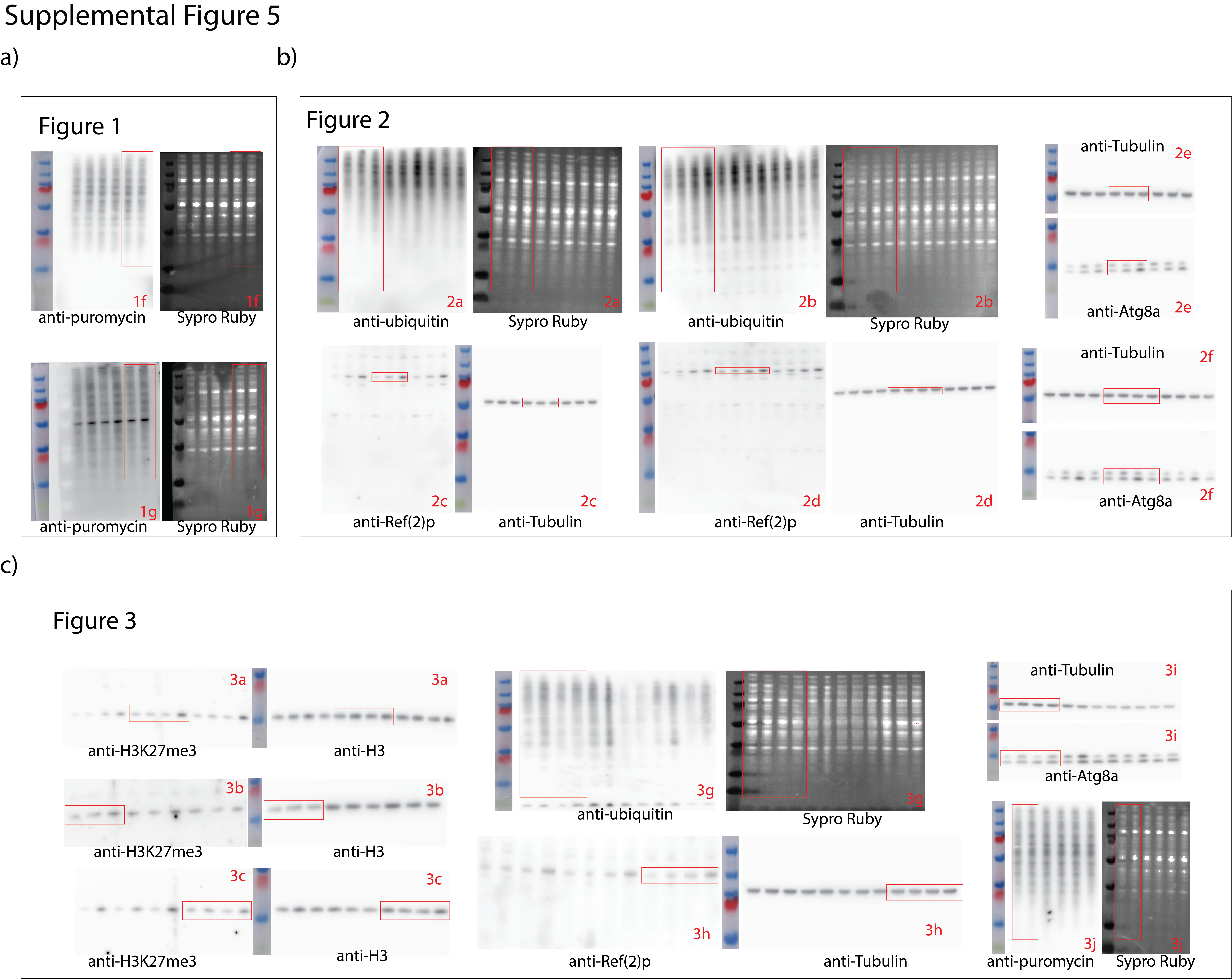
**

**
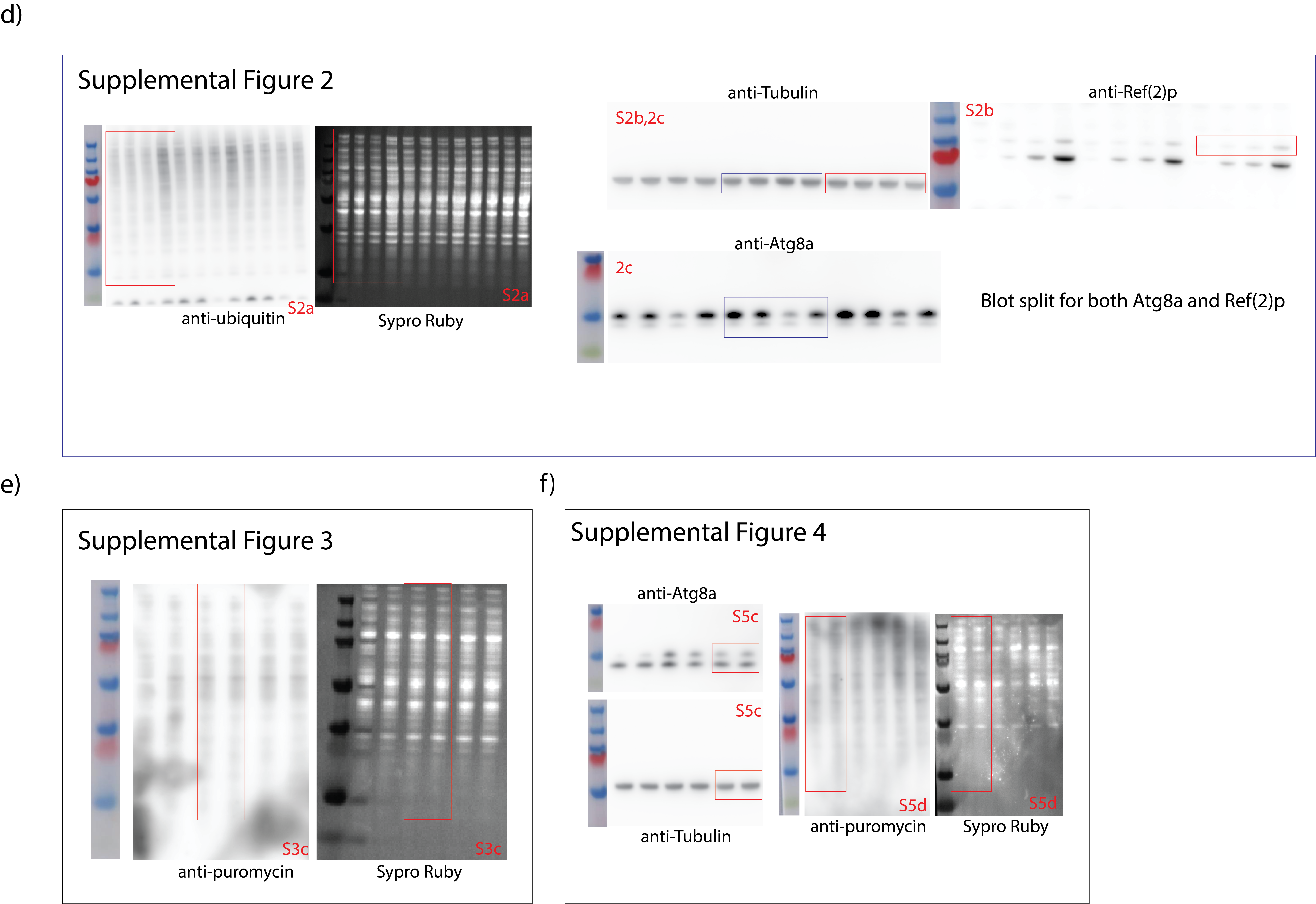
**

**Supplemental Figure 5. Uncropped lanes for all representative western immunoblots.**

**(a-f)** Each blot is labeled for its panel location. The red box around specific lanes denote the representative lanes used in each figure. The ladder is PageRuler Plus Prestained Protein Ladder.

**Supplemental Table 1: *Drosophila melanogaster* genotype information for figures**

| Figure | Genotype (name in this paper)^a^ |
| --- | --- |
| Figure 1 | Control |
| Figure 1 | *∆miR-34* |
| Figure 2 | Control |
| Figure 2 | *∆miR-34* |
| Figure 3 | Control |
| Figure 3 | *∆Utx* |
| Figure 3 | Rh1>SCA3trQ78 over + |
| Figure 3 | Rh1>SCA3trQ78 over ∆*Utx* or + |
| Figure 4 | Control |
| Figure 4 | *∆miR-34* |
| S Figure 2 | Control |
| S Figure 2 | *∆miR-34* |
| S Figure 2 | elavGAL4>Scramble-sp |
| S Figure 2 | elavGAL4>miR-34-sp |
| S Figure 3 | Control |
| S Figure 3 | *∆Utx* |
| S Figure 4 | Lst8 + Vehicle |
| S Figure 4 | Lst8 +RU486 |

^a^ Full Genotypes in Supplemental Table 2

**Supplemental Table 2: *Drosophila melanogaster* genotype information**

| Fly Line | ID | Genotype | Reference |
| --- | --- | --- | --- |
| Control | BDSC: 5905 | w1118 | Ma et al, 2018 |
| ∆*miR-34* (*miR-34* mutant) | - | w1118;;*miR-34* | Ma et al, 2018 |
| ∆*Utx* (*Utx* mutant) | - | w1118;*Utx*; | Ma et al, 2018 |
| Rh1>PolyQ | - | ;;rh1-gal4-1, P{w+mCUAS-Hsap\MJD.tr-Q78}c11.2/+ | Kennderdell et al, 2018 |
| Lst8 Upregulation | - | w*;daughterlessGS/UAS-Lst8.flag;Tm6b, Tb1 | This paper |
| UAS-Lst8* | BDSC: 53725 | w*; P{UAS-Lst8.flag}2; TM2/TM6B, Tb1 | - |
| daughterlessGS-GAL4* | - | ;da-GeneSwitch (II); | - |
| elavGAL4>Scramble-sp | - | elavGAL4;P{UAS-mCherry.mir-34.sponge.V2}attP40/+; P{UAS-mCherry.mir-34.sponge.V2}attP2/+ | This paper |
| elavGAL4>miR-34-sp | - | elavGAL4;P{y[+t7.7] w[+mC]=UAS-mCherry.scramble.sponge}attP40/+; P{y[+t7.7] w[+mC]=UAS-mCherry.scramble.sponge}attP2/+ | This paper |
| elav-gal4* | BDSC: 458 | elav-gal4;; | - |
| miR-34-sp* | BDSC: 61386 | w*; P{UAS-mCherry.mir-34.sponge.V2}attP40; P{UAS-mCherry.mir-34.sponge.V2}attP2/TM6B, Tb1 | Fulga et al, 2015 |
| Scamble-sp* | BDSC: 61501 | w[*]; P{y[+t7.7] w[+mC]=UAS-mCherry.scramble.sponge}attP40; P{y[+t7.7] w[+mC]=UAS-mCherry.scramble.sponge}attP2 | Fulga et al, 2015 |

*parent lines used for crosses

**Supplemental Table 3: Primer Sequences**

| Target gene | Assay | Forward Primer | Reverse Primer | Reference |
| --- | --- | --- | --- | --- |
| SCA3trQ78 | qPCR | CAGGACAGAGTTCACATCCATGT | GCCTTACCTAGATCACTCCCAAGT | Kennerdell et al, 2018 |
| ß-tubulin | qPCR | CATCCAAGCTGGTCAGTG | GCCATGCTCATCGGAGAT | Goodman et al, 2019 |
| Lst8 | qPCR | ATGGGGGACCAACAGCAGCT | GGTGTCCTGTCTAGCGCATT | This paper |

**Supplemental Table 4: Antibody Information**

| Antibody | Source and Catalog # | **Lot #** | **Method** | **Experiment Dilution** |
| --- | --- | --- | --- | --- |
| Primary Antibodies | | | | |
| mouse anti-Puromycin | Kerafast, 3RH11 | **210408** | **Western Immunoblot** | **1:1000** |
| mouse anti-ubiquitin | Cell Signaling Technologies, P4D1 | **17** | **Western Immunoblot** | **1:1000** |
| rabbit anti-Ref(2)p | Abcam, ab178440 | **GR3316044-1** | **Western Immunoblot** | **1:2500** |
| rabbit anti-GABARAP/Atg8a | Cell Signaling Technologies, E1J4E | **3** | **Western Immunoblot** | **1:5000** |
| rabbit anti-H3K27me3 | EMD Millipore, 07-449 | **3317006** | **Western Immunoblot** | **1:2500** |
| mouse anti-H3 | Cell Signaling Technologies, 96C10 | **10** | **Western Immunoblot** | **1:10000** |
| rat anti-HA | Roche, 3F10 | **11058700** | **Cryosection** | **1:100** |
| Secondary Antibodies | | | | |
| Goat anti-rabbit HRP | Jackson ImmunoResearch 111-035-144 | **127629** | **Western Immunoblot** | **1:5000 (1:2000 for rb anti-4E-BP1-phos)** |
| Goat anti-mouse HRP | Abcam, ab6789 | **N/A** | **Western Immunoblot** | **1:5000** |
| Goat anti-rat 594 | Invitrogen, A11007 | **1526627** | **Cryosection** | **1:200** |

**Supplemental File 1.**

DESeq2 analysis. Comparisons: 3d *miR-34* vs. 3d Control. 20d *miR-34* vs. 20d Control. 20d Control vs. 3d Control.

**Supplemental File 2.**

DESeq2 analysis. Comparisons: 20d Control vs. 3d Control. 50d Control vs. 3d Control. 50d Control vs. 20d Control.

**Supplemental File 3.**

GO Term Analysis on significantly upregulated and downregulated genes for the 3d *miR-34* vs. 3d control DEG list, 20d *miR-34* vs. 20d Control DEG lists, and for aging RNA-seq DEG lists. For each analysis (performed using Flymine v. 51 database) (Lyne et al., 2007), all GO Terms are listed.

**Supplemental File 4.**

Four proteostasis pathways were analyzed by curating gene lists from Flymine v. 51 (Lyne et al., 2007) (Translation, Chaperone, Autophagy, Proteosome).

**Supplemental File 5.**

213 predicted targets of *Drosophila miR-34* were identified using TargetscanFly database (Agarwal, 2018). All targets were mapped to DESeq2 DEG lists for 3d *miR-34* vs. 3d Control and 20d *miR-34* vs. 20d Control.

**Supplemental File 6.**

Detailed statistical test results for experiments in manuscript.
